# *Enterobacter cloacae* colonisation and infection in a neonatal intensive care unit: retrospective investigation of preventive measures implemented after a multiclonal outbreak

**DOI:** 10.1186/s12879-020-05406-8

**Published:** 2020-09-17

**Authors:** Alexandrine Ferry, Frank Plaisant, Christophe Ginevra, Yann Dumont, Jacqueline Grando, Olivier Claris, François Vandenesch, Marine Butin

**Affiliations:** 1 Hospices Civils de Lyon, Service de Néonatologie et Réanimation Néonatale, Hôpital Femme Mère 59 Boulevard Pinel, 69500 Bron, France; 2 Hospices Civils de Lyon, Institut des Agents Infectieux, Centre National de Référence des Staphylocoques, Groupement Hospitalier Nord, Lyon, France; 3 Université Claude Bernard, 4129 Villeurbanne, EA France; 4 CIRI, Centre International de Recherche en Infectiologie, Université de Lyon, Inserm U1111; Ecole Normale Supérieure de Lyon; Université Lyon 1; CNRS, UMR5308, Lyon, France

**Keywords:** *Enterobacter cloacae*, NICU, Outbreak, Cephalosporins, Biocleaning

## Abstract

**Background:**

*Enterobacter cloacae* species is responsible for nosocomial outbreaks in vulnerable patients in neonatal intensive care units (NICU). The environment can constitute the reservoir and source of infection in NICUs. Herein we report the impact of preventive measures implemented after an *Enterobacter cloacae* outbreak inside a NICU.

**Methods:**

This retrospective study was conducted in one level 3 NICU in Lyon, France, over a 6 year-period (2012–2018). After an outbreak of *Enterobacter cloacae* infections in hospitalized neonates in 2013, several measures were implemented including intensive biocleaning and education of medical staff. Clinical and microbiological characteristics of infected patients and evolution of colonization/infection with *Enterobacter* spp. in this NICU were retrieved. Moreover, whole genome sequencing was performed on 6 outbreak strains.

**Results:**

*Enterobacter* spp. was isolated in 469 patients and 30 patients developed an infection including 2 meningitis and 12 fatal cases. Preventive measures and education of medical staff were not associated with a significant decrease in patient colonisation but led to a persistent decreased use of cephalosporin in the NICU. Infection strains were genetically diverse, supporting the hypothesis of multiple hygiene defects rather than the diffusion of a single clone.

**Conclusions:**

Grouped cases of infections inside one setting are not necessarily related to a single-clone outbreak and could reveal other environmental and organisational problematics. The fight against implementation and transmission of *Enterobacter* spp. in NICUs remains a major challenge.

## Background

Hospitalized neonates, especially preterm infants, are at high risk of infection because of their immaturity and weak immune system. The rise of Gram-negative bacteria involved in neonatal infection is a major issue both because of the associated morbidity but also because of the emergence of multidrug resistant (MDR) strains [1–3]. In particular, the *Enterobacter cloacae* species is a Gram-negative bacteria responsible for nosocomial outbreaks in vulnerable patients in neonatal intensive care units (NICU) [2, 3].

The gut could constitute the reservoir of such infections because *Enterobacter* spp. establish early in the neonatal microbiota [4]. However, several outbreaks involving *Enterobacter cloacae* have been described over recent years, suggesting that the NICU environment or staff may also constitute the source of infection and transmission inside a NICU [2–5].

The aim of the present study was to evaluate over a 6 year-period the impact of preventive measures implemented after an outbreak of *Enterobacter cloacae* infections, on the gut colonisation and invasive infections with *Enterobacter cloacae* in one NICU.

## Methods

This retrospective study was conducted in the NICU of the Hôpital Femme-Mère-Enfant (Hospices Civils de Lyon) in Lyon, France from 1 January 2012 to 31 January 2018. During the summer 2013, several *Enterobacter cloacae* infections occurred and led to the implementation of preventive measures, including increasing precaution for sterile procedures, reminder of standard hand hygiene to the paramedical and medical staff, optimisation of the equipment circuit and maintenance. Moreover, intensive biocleaning, including fumigation and sterilisation of the ward, was performed in August 2013 and then once a year each following February. Finally, information was delivered to the medical staff with an emphasis on risks of using broad-spectrum antibiotics (effects on microbiota, emergence of resistance, etc.) and importance of using guidelines for empirical antibiotic prescriptions (decrease indications of cephalosporins, limit the use of broad-spectrum antibiotics including carbapenems and linezolid, early stop of antibiotics) (Additional file 1). All these measures were continued since 2013 and were intensified after a second outbreak in July 2014.

Data regarding isolation of *Enterobacter* spp. and antimicrobial susceptibility profiles were obtained from the microbiology department. In the studied NICU, screening for bacterial gut colonisation is routinely conducted weekly for all patients by collecting stools from diapers. *Enterobacter* spp. is identified when it is predominant in the stool (culture on non-selective agar) and/or if it grows on ChromiD BLSE® (bioMérieux, Marcy-l’Etoile, France). ChromiD BLSE® is a chromogenic agar containing an antimicrobial agent selecting only the strains that are resistant to third-generation cephalosporins (these strains were described as MDR strains in this study). Patients who had at least one stool culture positive for *Enterobacter cloacae* were considered as colonised. Those who had one positive blood and/or cerebrospinal fluid (CSF) culture associated with symptoms or signs of infection were considered as infected. In the study, we considered one positive stool sample per patient, per month for colonised patients and the first positive blood or CSF culture for infected patient.

Clinical data (baseline characteristics, maternal data, and for infected patients data concerning the episode of sepsis) were retrieved using ICCA software (Philips®, Suresne, France) which prospectively records medical information in the NICU.

The annual number of patients exposed to antibiotics during the study period was retrieved from ICCA software and from the database of the pharmacy. Data were obtained for carbapenems, cefotaxime and other beta-lactams (including amoxicillin, amoxicillin-clavulanic acid and penicillin G) from 2012 to 2017. Antibiotics use was represented as number of exposed patients for 1000 patient-day.

During the second outbreak in 2014, to evaluate the genetic relationships of *Enterobacter cloacae* strains from the NICU, whole genome sequencing (WGS) was performed on a subset of six strains including the four strains isolated in blood/CSF in July 2014 and two additional strains isolated from stools of non-infected neonates during the same period. Briefly, after DNA extraction, paired-end libraries of 100 bp read length were sequenced on an Illumina MiSeq platform. Genomes were assembled and compared with the 438 publicly available reference genomes of *Enterobacter cloacae* (ncbi/assembly) using popPUNK software [6].

## Results

**Fig. 1:**
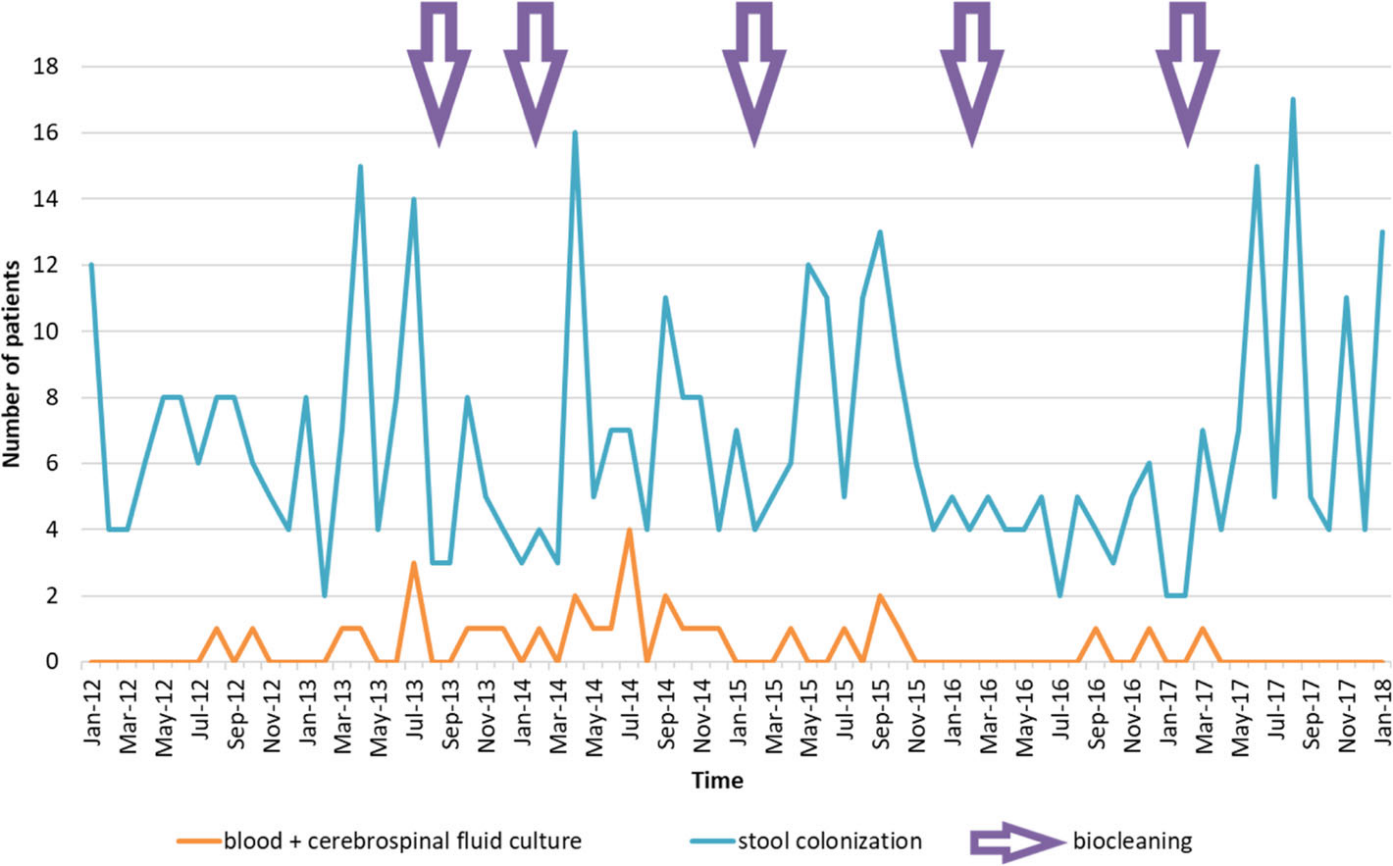
Number of *Enterobacter cloacae* positive samples (blood/CSF culture or stool culture) isolated each month in one NICU setting, between January 2012 and January 2018

During the study period, 30 patients developed an *Enterobacter cloacae* infection (including 22 in 2012–2014 and 8 in 2015–2018) and *Enterobacter cloacae* was isolated in 1341 stool cultures (469 patients). Interestingly, cases of infection did not necessarily occur during periods of high incidence of colonisation in the NICU and the annual intensive biocleaning of the ward showed only a moderate effect on the incidence of colonisation (Fig. 1).

**Fig. 2:**
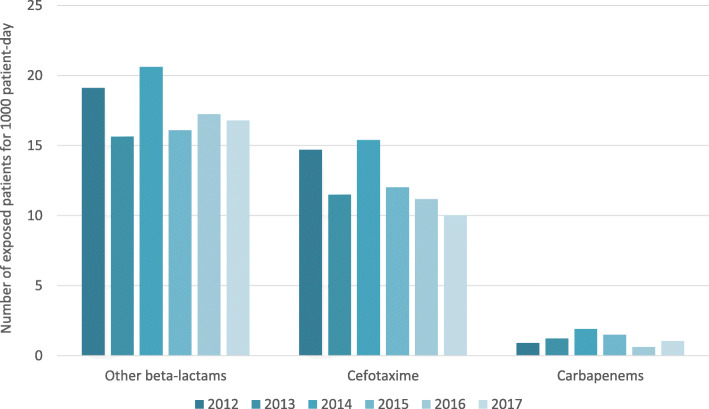
Number of patients exposed to three antibiotic classes administered in the NICU setting, for 1000 patient-day, between 2012 and 2017. “Other beta-lactams” include amoxicillin, amoxicillin-clavulanic acid and penicillin G. “Carbapenems” include imipenem and meropenem

After delivering guidelines to the medical staff in 2013–2014, a decrease of cefotaxime use (from 14.7 patients for 1000 patient-day in 2012 to 10.0 patients for 1000 patient-day in 2017), without a subsequent rise of carbapenem consumption was observed since 2014 (Fig. 2).

Because of the method of screening of *Enterobacter* spp. strains in stools, that identified preferentially and may overestimated the MDR ones, it was not relevant to describe the resistance profile of strains from colonization. Besides, a resistance to cephalosporins (including both phenotypes: cephalosporinase and extended spectrum beta-lactamase producers) was observed in 6.7% strains from blood cultures but none was resistant to carbapenem. Because a previous use of cephalosporin is a well-known risk factor for MDR phenotype acquisition [4], this was evaluated in the present study. Among the 30 infected patients, five mothers had received cephalosporin during delivery and two of their five neonates were colonized with a MDR strain. Moreover, among the 30 infected patients, three neonates had received cephalosporin during the first days of life. Among these three patients, one was colonized with a MDR strain and another was infected with a MDR strain.

The clinical characteristics of the 30 infected patients are summarized in Table 1. They were mainly preterm patients with a very low birth weight. Two patients developed meningitis. A total of 12 patients (40%) died after a median delay of 3 days following the beginning of sepsis. Among the 30 infected patients, one patient presented with an early-onset sepsis at day 1 of life that was considered as a maternal-fetal infection because the vaginal sample of his mother was positive for *Enterobacter cloacae*. Twenty-nine infections were considered as late-onset sepsis. Among them, 2 patients had undergone a surgery, which was considered as the origin of the infection because cultures of the surgical sites grew with *Enterobacter cloacae* (CSF positive after a ventricular-peritoneal shunt and peritoneal fluid positive after a gastrostomy placement).

**Table 1:** Characteristics of the 30 neonates infected with *Enterobacter cloacae* in one NICU setting between January 2012 and January 2018

Basic data	
sex ratio (M/F)	17/13
gestational age of birth (weeks)^a^	28.6 [24.3–41.1]
birth weight (g)^a^	930 [515–3770]
intra-uterine growth retardation, no (%)	10 (33.3%)
antenatal corticosteroids, no (%)	26 (86.7%)
caesarean, no (%)	25 (83.3%)
Apgar score (5 min)^a^	9 [1–10]
surgery before sepsis, no (%)	2 (6.7%)
*E. cloacae* in the vaginal sample of the mother, no (%)	1 (3.3%)
Sepsis data	
colonization with *E. cloacae* before sepsis, no (%)	4 (13.3%)
age at the onset of sepsis (days)^a^	7 [0–50]
fever, no (%)	11 (36.7%)
respiratory signs, no (%)	21 (70%)
hemodynamic signs, no (%)	27 (90%)
gastrointestinal signs, no (%)	15 (50%)
hyperglycaemia, no (%)	4 (13.3%)
central venous line at onset, no (%)	23 (76.7%)
CSF positive for *E. cloacae*, no (%)	2/18^b^ (11%)
Evolution	
length of stay^a,d^	47 [10–185]
necrotizing enterocolitis, no (%)	4 (13.3%)
death / death caused by sepsis, no (%)	12 (40%)/ 10 (33.3%)
delay between onset of sepsis and death (days)^a^	3 [0–26]
Management	
central venous line removal, no (%)	7/23^c^ (30.4%)
Antibiotics	
Carbapenem, no (%)	24 (80%)
Cephalosporins, no (%)	24 (80%)
Aminoglycosides, no (%)	30 (100%)
Ciprofloxacin, no (%)	5 (16.7%)
Piperacillin, no (%)	1 (3.3%)
duration of antibiotic (days)^a,d^	15 [10–34]

^a^Values are median [extremes]

^b^A bacterial analysis of CSF (cerebrospinal fluid) was performed in only 18 of the 30 patients

^c^Only 23 of the 30 patients patients had a central line at the onset of the sepsis

^d^Data are presented for the 18 patients who survived

The treatment was based on either cephalosporin or carbapenem. This treatment was administered at the onset of the sepsis treatment in 25 patients and was administered as a second-line treatment in 5 patients, who initially received antibiotics targeting Gram-positive bacteria (vancomycin). Among the five patients with delayed appropriate antibiotic, only one died. A central line was present in 23 patients at the onset of sepsis, and was removed in 7 patients because of the sepsis. The number of deaths was significantly higher in patients in whom the catheter was maintained (11 over 16 patients versus 1 over 7 patients, *p* = 0.02, Fisher’s Exact Test, two.sided).

**Fig. 3:**
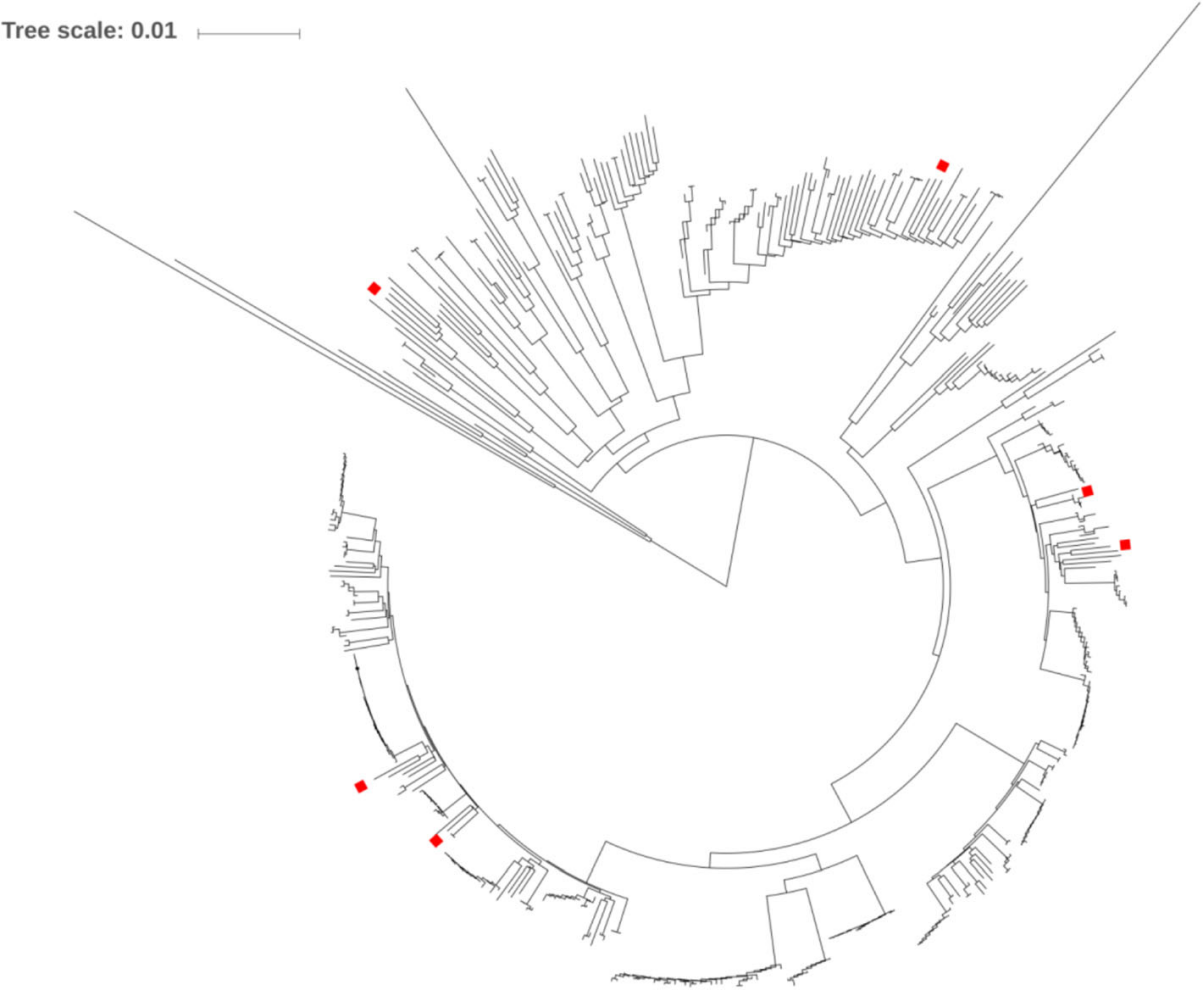
Phylogenetic tree based on whole genome sequencing comparison of six *Enterobacter cloacae* strains from the studied NICU (represented by red squares) with the 438 publicly available reference genomes of *Enterobacter cloacae* using popPUNK software

Finally, the WGS analysis showed high genomic distance between the six strains isolated during the second outbreak (July 2014) in the NICU (Fig. 3). Of note, these isolates did not constitute a separate cluster, but they were widely distributed along the phylogeny constructed with publicly available genomes.

## Discussion

In this study we report the clinical, microbiological and molecular characteristics of an *Enterobacter cloacae* outbreak in a NICU and the effect of subsequent preventive measures implemented in this NICU.

First, in a clinical point of view, the characteristics of the infected patients were similar to those described in the literature and none of the symptoms were either constant or specific of this infection, as already reported in previous similar studies [3]. The high mortality rate observed in our cohort (40%) was unexpected, since in the literature, it varies between 10% in a Taiwanese study [3], and 34% in a Brazilian study [7]. The high mortality rate herein cannot be explained by a delayed antibiotic administration. The rapid occurrence of death in infected patients suggests that intrinsic bacterial virulence factors may have been involved, but further studies are required to explore this hypothesis.

However, an additional hypothesis of this high rate of mortality is a part of the management of infected patient. Usually it is recommended to remove the central line in case of Gram-negative bacteraemia [8] but in our study, it was removed in only 30.4% patients. This conservative behaviour was explained because of the difficulty to insert a novel central venous line in vulnerable neonates and because of their frequent clinical instability requiring intravenous drugs. The retrospective design of our study does not allow to confirm causality between maintain of the catheter and death and it is possible that the decision to remove the catheter was more difficult in the most severe patients. However this conservation of catheter despite infection should be avoided.

In a pathophysiological perspective, it is known that *Enterobacter* spp. infection classically occurs after gut colonisation and translocation. Herein, 50% of the patients presented with gastrointestinal symptoms and 24% did not have central venous catheter at the onset of the sepsis, suggesting that translocation across the barrier gut could constitute the route of infection. However, only four infected patients were identified as colonised with *Enterobacter* spp. before the sepsis. The method and the frequency of stool screening (only once a week) could have underestimated colonisation, especially in case of colonisation with a non-resistant strain. However, the gastro-intestinal translocation is not the single way of invasion that could be involved in *Enterobacter* spp. infections. For example, herein two patients presented with a surgical site infection.

Furthermore, the environment could also constitute the reservoir of invasive strains [5]. The multi-clonal nature of the outbreak during July 2014 suggests the occurrence of multiple concomitant sepsis involving different clones in one NICU and supports the hypothesis of multiple hygiene defects and failures in the fight against nosocomial infections. That is why several preventive measures including an annual intensive biocleaning were implemented in the NICU. A decrease in the number of infected patients in this NICU was observed since the end of 2014. In the literature a previous study reported a good effectiveness of fumigation procedures in limiting *Enterobacter cloacae* infections in a NICU [5]. However other actions should have been implemented to limit the propagation of the outbreak. Patient cohorting is usually recommended but it was not possible due to the characteristics of the studied NICU (collective room with 7 beds). Moreover, other factors including overcrowding and/or understaffing have been reported as circumstances significantly associated with *Enterobacter* spp. sepsis [9] and thus could have favoured deficiencies in hygiene procedures during the outbreak. Data were not available to confirm this. Of note the importance of standard hand hygiene was emphasized during reminder to the paramedical and medical staff after the outbreak.

Another major factor involved in the selection of *Enterobacter* in NICU settings is the antimicrobial selective pressure. This has been demonstrated at the individual level: hospitalized neonates exposed to cephalosporins are at risk to colonize with antimicrobial-resistant Gram negative bacilli [10] and especially to *Enterobacter* spp. [4]. In that respect, in our study a previous use of cephalosporin was identified in some patients infected with a MDR *Enterobacter* spp. strain. Moreover the link between cephalosporin use and prevalence of *Enterobacter* colonization has also been demonstrated at the level of a NICU setting, hence modification of antimicrobial regimens can impact the prevalence of *Enterobacter* colonization in a NICU. A previous study inside a Brazilian NICU setting reported that the modification of first-line antibiotic for late-onset sepsis with restriction in the use of cephalosporin led to a significant decrease of cephalosporin-resistant *Enterobacter cloacae* [1]. Recently another study reported a 5 fold reduction of MDR *Enterobacter* colonization in a German NICU after implementation of antibiotic use recommendations including replacement of cephalosporins by alternative antibiotics when possible [11]. In the same way, guidelines about empirical antibiotic prescriptions were implemented in the studied NICU. As a result, the use of cephalosporins decreased in the studied NICU. This observation along with other experiences reported in the literature support the importance of local stewardships to reduce excessive antibiotics consumption. However the retrospective design of our study did not allow for exploring the impact of this reduction on the microbiological ecology of our NICU.

The study does have limitations. First, its retrospective nature does not allow us to ascertain causality and can induce bias, but this was limited by the prospective recording of medical information in the software. Second, during outbreaks no environmental samples were collected therefore we can only speculate whether or not the environment could constitute the reservoir of *Enterobacter cloacae*. Finally, due to technical constraints, it was not feasible to perform WGS on a large subset on strains, which limits the scope of the genomic findings.

## Conclusions

Neonatal sepsis involving *Enterobacter* spp. may be associated with a high rate of mortality. Grouped cases of infection inside one setting are not necessarily related to a single-clone outbreak and could reveal other environmental and organisational problematics. The fight against establishment and transmission of *Enterobacter* spp. and especially MDR strains inside a setting is a major challenge involving respect of standard hygiene, reinforcement of disinfection procedures and restriction of large spectrum antibiotic use.

## Supplementary information

**Additional file 1.**

## Data Availability

The genomic datasets supporting the results of this article are available from the European Nucleotide Archive under BioProject no PRJEB40194. The clinical datasets used and/or analysed during the current study are available from the corresponding author on reasonable request.

## Abbreviations

MDR: Multidrug resistant
NICU: Neonatal intensive care units
CSF: Cerebrospinal fluid
WGS: Whole genome sequencing
