# Supplementary material for: Enterobacter cloacae colonisation and infection in a neonatal intensive care unit: retrospective investigation of preventive measures implemented after a multiclonal outbreak
Source: BMC Infect Dis. 2020 Sep 17;20:682. doi: 10.1186/s12879-020-05406-8 (PMC7500001; doi:10.1186/s12879-020-05406-8)
Supplement: Supplementary file 1 [file 12879_2020_5406_MOESM1_ESM.odt]

Additional file 1. Guidelines for empirical antibiotic prescriptions

The present guidelines take into account the specific epidemiology of the study NICU and are not transferrable in other NICUs.

In case of early-onset sepsis, the first line antimicrobial regimen includes penicillin G and amikacin. Cephalosporin use should be limited to patients with a severe presentation including septic shock or meningitis. Cephalosporins can also be considered in case of a known maternal colonization with bacteria resistant to amoxicillin and susceptible to cephalosporins.

In case of late-onset sepsis, the first line antimicrobial regimen includes vancomycin and amikacin. Cephalosporin use should be limited to patients with gut disorders or enterocolitis.

Moreover in case of negative blood culture, antibiotics should be stopped after 48 hours, whatever is the clinical context. 

Finally the use of broad-spectrum antibiotics including carbapenems and linezolid should be limited to second-line treatment in case of documented multidrug resistant strain or in case of failure of first-line treatment.
